# Global Adoption, Promotion, Impact, and Deployment of AI in Patient Care, Health Care Delivery, Management, and Health Care Systems Leadership: Cross-Sectional Survey

**DOI:** 10.2196/70805

**Published:** 2025-10-22

**Authors:** Obinna O Oleribe, Andrew W Taylor-Robinson, Vetty R Agala, Olajide O Sobande, Ricardo Izurieta, Simon D Taylor-Robinson

**Affiliations:** 1 California State University, Dominguez Hills Carson, CA United States; 2 Office of the Director Centre for Family Health Initiative Orange, CA United States; 3 College of Health Sciences VinUniversity Hanoi Vietnam; 4 VinUniversity-University of Illinois Smart Health Center VinUniversity Hanoi Vietnam; 5 Center for Global Health Perelman School of Medicine University of Pennsylvania Philadelphia, PA United States; 6 Rivers State Contributory Health Protection Programme Port Harcourt Nigeria; 7 Nigerian Institute of Medical Research Foundation Lagos Nigeria; 8 Department of Surgery and Cancer Faculty of Medicine Imperial College London London United Kingdom

**Keywords:** artificial intelligence, health care delivery, health care system, leadership, management, patient care

## Abstract

**Background:**

Artificial intelligence (AI) is increasingly being integrated into health care, offering a wide array of benefits. Current AI applications encompass patients’ diagnosis, treatment, data mining, and more to enhance patient care and quality of life. It is also democratizing access to expert support by providing timely and accurate disease diagnoses, better clinical management, quicker drug discovery, improved disease prevention, big data management, and health protection.

**Objective:**

The aim of the study is to document AI adoption in health care, assess participants’ perception on its usefulness in the management of health care delivery and leadership of health care systems, and identify characteristics of early adopters.

**Methods:**

We conducted a worldwide cross-sectional survey across all 6 inhabited continents using a self-administered questionnaire developed with the Qualtrics electronic data collection tool. This was piloted and reviewed to ensure completeness, accuracy, acceptability, cultural sensitivity, and relevance. Respondents were recruited by individualized email, following identification from professional associations or organizations, professional networks, and social media. Data were analyzed using SPSS (IBM Corp), with results presented as narrative, charts, and tables.

**Results:**

In total, 506 health care professionals completed the survey. While 92.3% (467/506) of respondents believed that AI has a role in patient care and health care management, only 76.5% (300/392) were willing to support AI adoption and embedding in their organization. Although top managers are mainly responsible for adoption processes, staff training remains low. AI is currently used mostly for diagnosis, patient care, and precision medicine. These uses of AI will continue in the near future, but in different ways. AI adoption was highest in Europe and lowest in Africa. Black or African American people were more likely to support AI adoption than White and Asian people. Poor knowledge of AI, fear of job loss, and resistance to change were the top barriers to AI adoption and embedding.

**Conclusions:**

AI use in health is global, but the adoption rate varies by geography and individual characteristics. AI adoption communication by executive health care management is poor, as is the level of training of health care staff. To improve AI adoption, management should improve communication with their teams, provide training on AI to their workers, and help individuals understand how AI works. Barriers such as ethical issues around data ownership and use should be addressed. African organizations should be proactive and invest in AI adoption early, so that they are not left behind in the AI revolution.

## Introduction

Artificial intelligence (AI) is increasingly being integrated into health care, offering a wide array of benefits [1-5]. Current AI applications encompass patients’ diagnosis, treatment, data mining, and more to enhance patient care and quality of life. Recent breakthroughs in AI and machine learning have led to the building of reliable and safe AI systems capable of handling the complexity of health care support in disciplines such as cardiology, radiology and oncology, health information management, patient care, and workforce development. AI fast tracks results in emergency rooms, trauma units, supply chain systems, and radiology and pathology units, for example. The impact is to minimize deficiencies in human resources, provide considerable benefits for patient safety and quality of care, alleviate clinician burnout, and decrease health care costs over time.

These gains offer great transformative potential, enabling acceleration of processes in diverse areas of health care [1-3]. Furthermore, AI is democratizing access to expert support by providing timely and accurate disease diagnoses, better clinical management, quicker drug discovery, improved disease prevention, big data management, and enhanced health protection [4,6]. In addition, 23% of health care executives in the United States believe that AI and machine learning are very effective at improving clinical outcomes [5].

The potential of AI to optimize resource use and boost productivity underscores its critical role in health care management and patient care. Building on this promising start necessitates striking a balance between technical advancements and management of ethical considerations. Responsible deployment of AI applications by the health care sector can lead to automated services, effective care delivery, and improved patient outcomes. This transformation requires policy support through strong governance, and for ethical concerns and regulatory issues to be addressed.

The current use of AI complements and boosts human resources and has a far-reaching impact in health management, health care worker training, and health services. Its full adoption will enhance leadership and management experiences in the delivery of care while supporting current functions and future innovations in the health care sector [7,8]. To ensure that health care workers are adequately equipped, skilled, and trained to use AI proficiently in health management and clinical practice, health care managers, educators, and providers must play a more proactive role in the ongoing digital revolution to ensure its effective and regular use [9-11]. Furthermore, health care leaders should actively promote the translation of innovation and research findings into health care management and delivery practices [11,12]. This will help revolutionize health care management, improve resource use, advance precision medicine, institutionalize operational efficiency, automate processes, and maximize predictive analytics [13].

Understanding AI adoption, progression, characteristics of early adopters, barriers to adoption, the extent to which and why individuals support AI adoption, and the role played by different levels of health management or leadership will inform future AI-related programs and policies. This will enable the global health care sector to adopt new management or leadership styles and organizational structures to harness the potential of AI effectively [13]. This study aimed to garner viewpoints from across the world on AI adoption in health care, its usefulness in patient care and health management or leadership, and the characteristics of early AI adopters.

## Methods

### Study Design

We conducted a worldwide cross-sectional survey using a self-administered questionnaire developed with the Qualtrics electronic data collection tool, targeting senior health care professionals for their opinions on the use of AI in the health care sector. The questionnaire included sections on AI adoption, deployment, use, benefits and barriers to AI adoption, as well as basic, anonymized demographic information of the participants (Multimedia Appendix 1). We piloted and reviewed the questionnaire to ensure completeness, accuracy, acceptability, cultural sensitivity, and relevance. The questionnaire and subsequent data analysis complied with the protocol and checklist outlined in CHERRIES (Checklist for Reporting Results of Internet E-Surveys) [14] (Multimedia Appendix 2).

The study population comprised health care executives, health care leaders, and service providers drawn from the 6 inhabited continents of the world (North and South America, Asia, Africa, Australia or Oceania, and Europe). We used a population prevalence of 23% and a confidence level of 95% to arrive at a sample size of 273 [5,15].

Health care executives and providers were identified through a convenience sampling technique of professional associations or organizations, social media platforms, and professional networks. The survey was sent to over 500 health care professionals, using their personalized email addresses. We also used professional networks to distribute the questionnaire, and anyone who received the questionnaire link could participate in the study. Participants were informed in the invitation email of the nature of the study, the length of time necessary to complete the questionnaire (less than 10 minutes), and the identity of the principal investigator (OOO). Participants were also informed that fully anonymized information would be collected and stored for 6 months behind a firewall and that no personal, traceable, or identifiable data would be collected. A link was provided in the email to the questionnaire, which required a “1-time only” password to prevent multiple completions of the questionnaire by individual participants.

Participation was voluntary with no incentives offered, and data collection occurred over a 7-week period from October 1 to November 19, 2024. The questionnaire was formatted over 16 pages with 1 to 2 questions per page and hosted on the Qualtrics website for the duration of the study. Participants were able to check for completeness and could review their answers using a “back button.” If participants were unsure or unwilling to disclose their responses, options including “not sure,” “not applicable,” or “prefer not to say” were available. According to the Qualtrics assessment, the study had a 99% completion rate for those persons who received the email, accessed the link, and started to complete the questionnaire.

After closure of the survey, data analysis was performed on anonymized, submitted questionnaires using SPSS (version 27; IBM Corp) and Microsoft Excel. Data were uploaded automatically by Qualtrics, rather than being uploaded manually for analysis. Descriptive and comparative analyses were conducted including frequencies, percentages, and chi-squares. A *P* value of <.05 was considered significant. To maintain participants’ anonymity, data were aggregated prior to analysis. Results are presented in tables, charts, and as narrative formats for clarity and comprehension.

### Ethical Considerations

Ethics approval was received from the California State University, Dominguez Hills Institutional Review Board (IRB) following an IRB approval application (CSUDH IRB-FY2024-66). An information sheet was provided before completion of the questionnaire, informing participants of the survey duration (up to 10 minutes), how the data were to be used, and that participation was optional and asking whether each participant was willing to proceed on that basis. Informed consents were obtained from participants before access to the questionnaire was given. Before completing the study, participants consented to their responses being used in an anonymized, aggregated fashion for medical research purposes and for subsequent secondary data analysis only. Only anonymized data were collected with no traceable information to the individual participants. The data were kept behind an institutional firewall at California State University, Dominguez Hills. Prior parental informed consent was requested in the event that a participant was under the age of consent. However, there was no participant younger than 18 years of age included in the study. No compensation was given for participating in this study.

## Results

### Overview

A total of 506 health care leaders and service providers participated in the survey. Most participants were drawn from Africa, North America, and Europe. The sex distribution was nearly equal, with the male population accounting for 47.8% (181/379) and the female population for 49.1% (186/379) of respondents. The rest were nonbinary or third sex (2/379, 0.5%), others (1/379, 0.3%), and those who preferred not to disclose their sex (9/379, 2.4%). Most participants (265/376, 70.5%) were between the ages of 30 and 59 years. Regarding level of education, 87.3% (331/379) were university graduates, with 71.5% (271/379) holding an advanced degree—a master’s degree (146/379, 38.5%) or a doctorate (125/379, 33%)—as their highest qualification.

In terms of professional experience, respondents were evenly distributed, with similar proportions having less than 10 years and 25 years or more. Approximately half (195/377, 51.7%) were affiliated with federal, state, or college or university systems, and 44.7% (226/506) worked in the health care services, while 28.3% (143/506) were affiliated with the educational sector. Over half (200/373, 53.6%) of the respondents were identified as Black or African American people. Geographically, the majority were based in Africa (159/373, 42.6%), North America (130/373, 34.9%), or Europe (49/373, 13.1%), as outlined in Table 1. Black or African American constituted the largest population of respondents (200/373, 53.6%), followed by White (55/373, 14.7%).

To the question “Do you think AI has any role or usefulness in health care practice and management?” 92.3% (467/506) responded Yes. Of these, 58.7% (254/433) were of the view that AI is very (179/433, 41.3%) or extremely (75/433, 17.3%) important. In the near future, 79.8% (375/470) were of the view that AI will be very (199/470, 42.3%) or extremely (176/470, 37.4%) useful (Table 2).

A total of 95.6% (173/181) of male participants, 91.9% (171/186) of female participants, 95.6% (154/159) of those from Africa, 93.9% (46/49) of those from Europe, 87.7% (114/130) of those from North America, 95.5% (191/200) of those who identified as Black or African American, 92.7% (51/55) of White, and 86.7% (26/30) of Hispanic or Latino participants stated that AI was useful for health management and patient care. There was a significant difference in the responses of male participants versus female participants (*P*<.001), organizational affiliations (*P*=.01), and continent of residence or operation (*P*=.03; Tables 3-6). Similarly, 87.8% (173/181) of male, 75.3% (140/186) of female, African (147/159, 92.5%), Black or African American (178/200, 89%), European (38/49, 77.6%), Hispanic or Latino (19/30, 63.3%), North American (87/130, 66.9%), and White (40/55, 72.7%) participants were of the view that AI is very or extremely useful in health; however, only sex (*P*=.01) and the continent of residence or operation (*P*=.001) showed a significant difference.

**Table 1:** Demographic characteristics of respondents.

Description		Values, n (%)
**Sex** **at birth (n=379)**		
	Male	181 (47.8)
	Female	186 (49.1)
	Nonbinary or third sex	2 (0.5)
	Others	1 (0.3)
	Prefer not to say	9 (2.4)
**Age in completed years (n=376)**		
	<20	4 (1.1)
	20-29	45 (12)
	30-39	89 (23.7)
	40-49	104 (27.7)
	50-59	72 (19.1)
	>60	62 (16.5)
**Highest educational qualification (n=379)**		
	High school diploma or General Educational Development	26 (6.9)
	Bachelors	60 (15.8)
	Masters	146 (38.5)
	Doctorate	125 (33)
	Other	17 (4.5)
	Prefer not to specify	5 (1.3)
**Length of experience in health care (years; n=372)**		
	<5	85 (22.8)
	5-9	54 (14.5)
	10-14	61 (16.4)
	15-19	40 (10.8)
	20-24	50 (13.4)
	>25	82 (22)
**Affiliation (n=377)**		
	Federal or state government facility or institution	100 (26.5)
	County or local government facility or institution	24 (6.4)
	College or university	95 (25.2)
	Nonprofit or public charity	56 (14.9)
	Private facility or institution	67 (17.8)
	Other	35 (9.3)
**Professional track (select all that apply)^a^ (n=506)**		
	Health care services	226 (44.7)
	Health education	143 (28.3)
	Health informatics	45 (8.9)
	Health technology	32 (6.3)
	Health administration or management	120 (23.7)
	Other	67 (13.2)
**Race or ethnicity (n=373)**		
	Arab or Middle-Eastern	6 (1.6)
	Asian (East and South)	25 (6.7)
	Black or African American	200 (53.6)
	Hispanic or Latino or Latinx	30 (8)
	Native American or Alaskan Native	3 (0.8)
	Pacific Islander or Hawaiian	1 (0.3)
	White	55 (14.7)
	Mixed	17 (4.6)
	Other	19 (5.1)
	Prefer not to say	17 (4.6)
**Location or residence (n=373)**		
	Africa	159 (42.6)
	Europe	49 (13.1)
	Asia	19 (5.1)
	North America	130 (34.9)
	South America	8 (2.1)
	Australia or Oceania	8 (2.1)

^a^Participants were able to select multiple options.

**Table 2:** Artificial intelligence (AI) relevance, use, and adoption in organizations.

Description			Yes, n (%)
**Do you think AI has any role or usefulness in health care practice and management? (n=506)**			
	Yes	467 (92.3)	
	No	13 (2.6)	
	Neither	26 (5.1)	
**If yes, how useful do you think AI is now in health care management and practice? (n=433)**			
	Not useful	9 (2.1)	
	Slightly useful	61 (14.1)	
	Moderately useful	109 (25.2)	
	Very useful	179 (41.3)	
	Extremely useful	75 (17.3)	
**In the future, how useful do you think AI will be in health care management and practice? (n=470)**			
	Not useful	4 (0.9)	
	Slightly useful	20 (4.3)	
	Moderately useful	71 (15.1)	
	Very useful	199 (42.3)	
	Extremely useful	176 (37.4)	
**Have you had any formal exposure or training in AI? (n=470)**			
	Yes	142 (30.2)	
	No	302 (64.3)	
	Not sure	26 (5.5)	
**If yes, what kind of training or exposure did you have? (Select all that apply)^a^ (n=142)**			
	Basic orientation	89 (62.7)	
	Training in AI use in patient care (diagnosis, treatment, laboratory services, etc)	24 (16.9)	
	Training in AI use in management and leadership	43 (30.3)	
	Training in technical aspects of AI	43 (30.3)	
	Other forms of AI training	33 (23.2)	
**Has your institution or organization adopted or begun the process of AI adoption, adaptation, and use? (n=457)**			
	Yes, we have adopted AI	60 (13.1)	
	Yes, we will adopt AI	36 (7.9)	
	Yes, we are beginning to think about adopting AI	107 (23.4)	
	No, we have not started adopting AI	180 (39.4)	
	Do not know	74 (16.2)	
**Has your organization trained anyone on AI use? (n=445)**			
	Yes	93 (20.9)	
	No	197 (44.3)	
	Do not know or not sure	155 (34.8)	
**If staff have been trained, who benefited from the training? (n=210)**			
	Executive leadership or top level	33 (15.7)	
	Management staff or mid-level	47 (22.4)	
	Operational staff	58 (27.6)	
	IT staff	44 (21)	
	Other	24 (11.4)	
	Prefer not to say	4 (1.9)	

^a^Participants were able to select multiple options.

**Table 3:** Artificial intelligence (AI) adoption and institutional capacity development by ethnicity and race.

Description	Race or ethnicity				Chi-square (*df*)		*P* value
	Black or African American (200/373, 53.6%); yes, n (%)	White (55/373, 14.7%); yes, n (%)	Hispanic or Latinx (30/373, 8%); yes, n (%)				
AI has a role in health care practice and management	191 (95.5)	51 (92.7)	26 (86.7)	21.8 (20)		.35	
AI will be very to extremely useful in health care management and practice	178 (89)	40 (72.7)	19 (63.3)	52.3 (40)		.09	
Have had formal exposure or training in AI	69 (34.5)	15 (27.3)	6 (20)	37.6 (20)		.01	
The organization has adopted AI	17 (8.5)	12 (21.8)	4 (13.3)	40.6 (40)		.45	
The organization has trained someone on AI use	32 (16)	14 (25.5)	4 (13.3)	13.3 (20)		.87	
The participant will support AI adoption and embedding	168 (84)	39 (70.9)	17 (56.7)	35.5 (20)		.01	

**Table 4:** Artificial intelligence (AI) adoption and institutional capacity development by geographical location.

Description	Continent				Chi-square (*df*)		*P* value
	Africa (159/373, 42.6%); yes, n (%)	Europe (49/373, 13.1%); yes, n (%)	North America (130/373, 34.9%); yes, n (%)				
AI has a role in health care practice and management	154 (96.9)	46 (93.9)	114 (87.7)	20.1 (10)		.03	
AI will be very to extremely useful in health care management and practice	147 (92.5)	38 (77.6)	87 (66.9)	44.5 (20)		.001	
Have had formal exposure or training in AI	46 (28.9)	15 (30.6)	43 (33.1)	13.4 (10)		.20	
The organization has adopted AI	12 (7.5)	12 (24.5)	21 (16.2)	41.6 (20)		.003	
The organization has trained someone on AI use	21 (13.2)	12 (24.5)	30 (23.1)	27.8 (10)		.002	
The participant will support AI adoption and embedding	145 (91.2)	41 (83.7)	76 (58.5)	53.6 (10)		<.001	

**Table 5:** Artificial intelligence (AI) adoption and institutional capacity development by biological sex.

Description	Sex				Chi-square (*df*)		*P* value
	Male (181/379, 47.8%); yes, n (%)	Female (186/379, 49.1%); yes, n (%)	Other (12/379, 3.2%); yes, n (%)				
AI has a role in health care practice and management	173 (95.6)	171 (91.9)	7 (58.3)	36.4 (8)		<.001	
AI will be very to extremely useful in health care management and practice	159 (87.8)	140 (75.3)	4 (33.3)	31.7 (16)		.01	
Have had formal exposure or training in AI	55 (30.4)	56 (30.1)	3 (25)	3.0 (8)		.94	
The organization has adopted AI	24 (13.3)	22 (11.8)	3 (25)	26.1 (16)		.05	
The organization has trained someone on AI use	38 (21)	31 (16.7)	3 (25)	9.0 (8)		.34	
The participant will support AI adoption and embedding	163 (90.1)	123 (66.1)	4 (33.3)	71.3 (8)		<.001	

**Table 6:** Comparison of key indications with chi-square analysis.

Description	Sex at birth		Age			Education			Experience			Affiliation			Race or ethnicity			Continent	
	Chi-square (*df*)	*P* value	Chi-square (*df*)	*P* value	Chi-square (*df*)		*P* value	Chi-square (*df*)		*P* value	Chi-square (*df*)		*P* value	Chi-square (*df*)		*P* value	Chi-square (*df*)		*P* value
AI^a^ has a role in health care practice and management	36.4 (8)	<.001	12.1 (10)	.28	3.2 (10)		.98	14.4 (10)		.16	22.5 (10)		.01	21.8 (20)		.35	20.1 (10)		.03
AI will be very to extremely useful in health care management and practice	31.7 (16)	.01	24.9 (20)	.21	28.7 (20)		.09	18.1 (20)		.58	30.6 (20)		.06	52.3 (40)		.09	44.5 (20)		.001
Have had formal exposure or training in AI	3.0 (8)	.94	30.4 (10)	<.001	19.8 (10)		.03	43.0 (10)		<.001	11.0 (10)		.36	37.6 (20)		.01	13.4 (10)		.20
The institution has adopted AI	26.1 (16)	.05	35.7 (20)	.01	61.4 (20)		<.001	28.1 (20)		.11	41.8 (20)		<.001	40.6 (40)		.45	41.6 (20)		.003
The organization has trained someone in AI use	9.0 (8)	.34	11.9 (10)	.29	29.7 (10)		<.001	29.1 (10)		.001	43.1 (10)		<.001	13.3 (20)		.87	27.8 (10)		.002
The participant will support AI adoption and embedding	71.3 (8)	<.001	52.2 (10)	<.001	22.1 (10)		.01	27.4 (10)		.002	16.1 (10)		.20	35.5 (20)		.02	53.6 (10)		<.001

^a^AI: artificial intelligence.

On training and exposure to AI, the study revealed that 30.2% (142/470) of the respondents have had formal exposure or training to AI, the majority (89/142, 62.7%) had a basic orientation, 13.1% (60/457) of institutions or organizations have adopted AI in any form, and 20.9% (93/445) have trained management, operational, and IT staff on AI use. Approximately equal percentages of male (55/181, 30.4%) and female (56/186, 30.1%) participants have been exposed to AI technology. However, more people were exposed to AI in North America (43/130, 33.1%) and Europe (15/49, 30.6%) than in Africa (46/159, 28.9%), but this difference was not statistically significant (*P*=.20). In addition, more Black or African American (69/200, 34.5%) and White (15/55, 27.3%) participants were exposed to AI than Hispanic or Latino (6/30, 20%) participants, and this was statistically significant (*P*=.01). Furthermore, more male participants reported that their organizations had trained people in AI (38/181, 21% vs31/186, 16.7%), but this difference was not significant (*P*=.03). European (12/49, 24.5%) and North American (30/130, 23.1%) participants reported more training than African participants (21/159, 13.2%), and this difference was statistically significant (*P*=.002). However, reported training across racial or ethnic groups was not statistically significant (*P*=.87).

**Figure 1:**
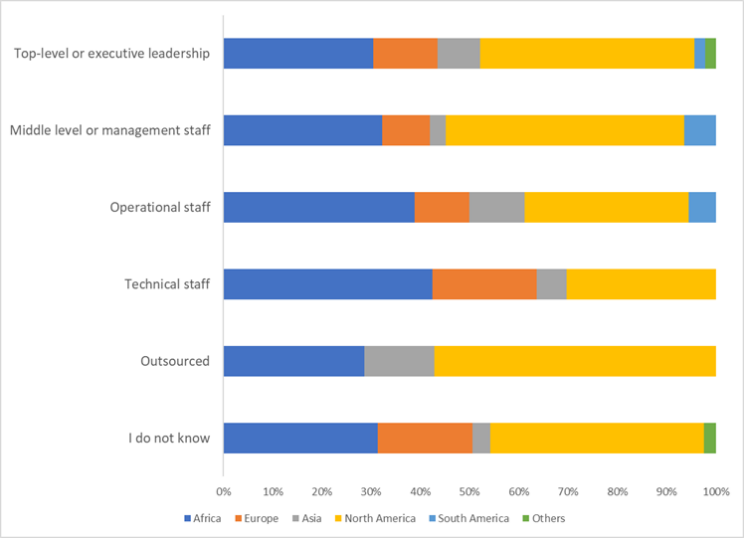
Artificial intelligence adoption proponents in health care organizations.

Among all respondents, 76.5% (300/392) will support AI adoption and embedding in their organization. AI adoption is led mainly by top executive leadership. However, other levels of leadership play significant roles, as shown in Figure 1.

A total of 38.6% (102/264) of respondents did not know who was or is leading the artificial intelligence adoption process in their organization. More organizations with male respondents have adopted AI (24/181, 13.3% vs 22/186, 11.8%), female respondents (*P*=.05). Of note, 24.5% (12/49) of European, 16.2% (21/130) of North American, but only 7.5% (12/159) of African organizations have adopted AI (*P*=.003; Tables 4 and 5). The continent of residence or operation had the highest levels of significance in all parameters except formal exposure to AI (α=.20; Table 6).

### The Use of AI

**Figure 2:**
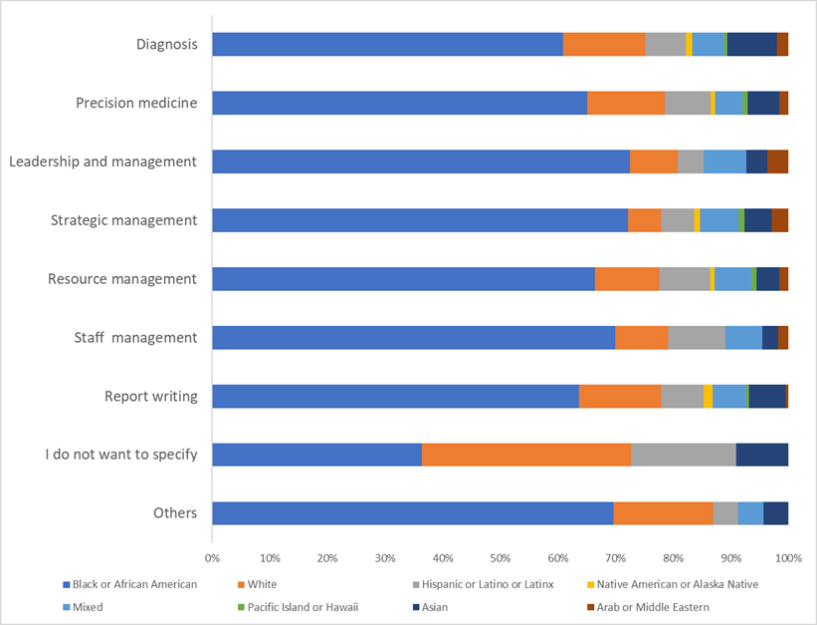
Uses of artificial intelligence in the health care industry. Participants were able to select multiple options.

AI is used mostly in diagnosis (247/506, 48.8%), report writing (230/506, 45.5%), and patient care (205/506, 40.5%). Other areas of high use are in precision medicine (160/506, 31.6%), resource management (156/506, 30.8%), staff management, and leadership and management (137/506, 27.1% each; Figure 2).

Similarly, in the coming years, it was predicted that AI will still be most relevant in diagnosis (120/407, 29.5%), patient care (75/407, 18.4%), precision medicine (65/407, 16%), and report writing (58/407, 14.3%). Respondents also identified other uses of AI. These include (among several others) marketing, counseling, emergency preparedness and response, customer services, real-time data management, drug discovery and development, modeling and predictive analytics, a tool for triage at the first point of contact, accurate billing and fraud detection, across-board process sequencing, advancing science and policy, monitoring air quality within the health care system, data management (eg, data access, analysis, processing, and report development), assessment (preanalytical, analytical, and postanalytical stages in health care), research (such as cancer research and treatment), environmental sanitation (such as cleaning toilets and emptying bedpans), customer services (such as customer complaints and solutions and generating referral codes), supply chain management, outreach programs, disease surveillance (including mapping, prediction, and preventive medical interventions), documentation, health care education (including staff training), communication and information management (including referrals, collaboration, and electronic health records), report writing (including minutes of meetings), insurance discoveries, data to inform decisions in most domains, health care advocacy, streamlining and standardizing systems (including processes, charting, referrals, and billing), schedule management (for employees and clients), claims and billing, disease surveillance and epidemic early warning, strategy development and leadership and management in global health, staff management (including hiring, training, and evaluation), prediction and management of pandemics and other emergency situations, procurement and purchasing tenders, strategic management, surveys, and community outreach.

AI use in patient care also includes deployment in surgeries (including robotic surgical equipment), preventive health care, patient follow-up or tracking, virtual health, remote patient monitoring, clinical decision support, patient engagement, detecting adverse events in inpatient and outpatient care, care coordination, chart making, blood and blood products delivery in rural and underserved communities, fast diagnosis and prompt treatment including gene therapy and infection control measures, patient reminders to adhere to their medication and appointment schedules, registration and recording, and prescription management.

In this regard, some respondents commented, “Virtually all aspects with no exception,” “Every aspect including management,” “Every aspect of health care,” “Everywhere,” “In all areas of health care,” and “In all areas of health care delivery and improvement.”

### Barriers to AI Use

**Figure 3:**
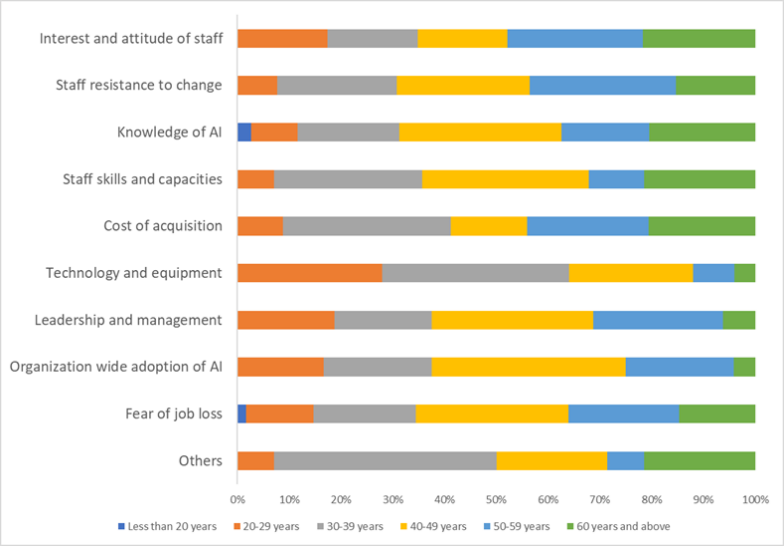
Barriers to AI adoption as identified by respondents. Participants were able to select multiple options. AI: artificial intelligence.

Knowledge of AI (121/402, 30.1%), fear of job loss (64/402, 15.9%), and staff resistance to change (39/402, 9.7%) were the top barriers identified by respondents against AI adoption (Figure 3). Other barriers are the cost of acquisition and inadequate staff skills (Table 6).

Apart from the itemized barriers, respondents further identified other barriers including poor access to relevant training, hallucination and possible AI inaccuracy, unreliable results, annoying responses in patient care or calls, clinical errors, confidentiality issues, inequitable, patient preference (such as patients not wanting computers or “bots” to talk to them), quality and trustworthiness of AI, grant writing, validity of results, and religious beliefs. Some respondents said, “All options are important barriers.”

Although three-quarters of people were ready to support AI adoption (300/392, 76.5%), 23.5% (92/392) will either not support AI adoption (13/392, 3.3%) or not be sure (79/392, 20.2%). Participants who were unwilling to support AI adoption listed security reasons, lack of in-depth understanding of how AI works, privacy issues, cost, fear of job loss, inaccuracy of results, uncarted future, fear of change, lack of human emotions with AI, fear of AI displacing humanity in patient care, distrust of AI, ignorance of AI and use in health care, patient safety, inferiority to human care, privacy risks, and cost of adoption among all others as their top reasons. A respondent said, “Until it becomes enforced, there will be no need for it.”

## Discussion

### Principal Findings

The adoption, promotion, impact, and deployment of AI in health leadership and management are a multifaceted process that encompasses various dimensions of health care delivery. The results presented here demonstrate the high importance of AI across all segments, but the adoption of AI and training of workers in AI is low and should be improved upon. A significant proportion of respondents were of the view that AI is already very useful in health care management and patient care and that its value will increase in the future.

Among our survey respondents, exposure to or training in AI was very low (142/470, 30.2%), the majority of which has been basic orientation (89/142, 62.7%). This indicates a serious gap in AI-related professional development that needs to be filled. Given this finding, it is not surprising that participants highlighted the knowledge (and, where relevant, skills) gap as the top barrier to adoption of AI. It is axiomatic, therefore, that if the health care sector wants to improve AI adoption, health care workers and managers must be trained in AI use to a level appropriate for their role.

Similarly, only 13.1% (60/457) of all institutions participating in this survey have adopted AI to date, while 7.9% (36/457) are considering its implementation soon. However, a further 23.4% (107/457) disclosed that they are starting to think about adopting AI. This is not unexpected, as AI is still new, and many people (including some participants) are unaware of its usefulness, skeptical of its precision, and uninformed about its range of applications. Adoption is also low due to key barriers such as fear of job loss, staff resistance to change, and cost, perceived or real, of adoption.

### Comparison to Prior Work

Herein, 20.9% (93/445) of respondents said that their organization had trained someone in the use of AI. This 1 in 5 participation rate compares to the 11.4% reported in a recently published survey on AI chatbot use by medical researchers published in 2024 [16]. The almost doubling may be due to a combination of many factors, among others including the time difference—a year between the 2 studies, greater awareness, increased adoption, and a reflection that chatbots capture only a small proportion of AI uses in health care. Moreover, in the 2024 study, 69.7% of surveyed individuals expressed interest in further training, whereas 79.5% of participants in our survey would support AI adoption in their organization [16].

The drive to adopt AI in the health care sector is primarily led by top leadership and management, followed by technical staff. Very few participating organizations (8/264, 3%) outsourced this process. Therefore, promoting AI technologies was influenced by both top-down and bottom-up approaches. However, more than 38% (102/264) of respondents did not know who was leading the AI movement in their organization. Such a lack of awareness calls for better communication between managers and operational or field staff. If not addressed, it may result in change implementation failure, as it is widely recognized that poor communication is the most common cause of change failure in change implementation [17].

The integration of AI technologies in health care has the potential to transform health systems, improve patient outcomes, and enhance operational efficiencies [1-5]. Our study confirms previous reports, showing that AI is useful across several domains of health management and patient care. These include diagnosis and patient care, data management and review, staff and resource management, research and pipeline analysis, and report writing, a portfolio that is predicted to expand in the coming years. AI was most relevant to in-patient care, diagnosis, precision medicine, and report writing, corroborating previous findings [18,19].

Among our survey respondents, more Black or African American (drawn from Africa, Europe, and North America) respondents were trained or exposed to AI (69/200, 34.5%) compared to White (15/55, 27.3%) and Hispanic or Latino (6/30, 20%) respondents, and this difference is significant (*P*=.01). However, geographically, fewer participants from Africa (46/159, 28.9%) have had formal training or exposure to AI compared to participants from Europe and North America; fewer organizations in Africa (12/159, 13.2%) have trained their staff in AI compared to organizations in North America (30/130, 23.1%) and Europe (12/49, 24.5%). Furthermore, AI adoption is lowest in Africa (12/159, 7.5%) but highest in Europe (12/49, 24%). This finding is not surprising, as historically, Africa has faced challenges in adopting new technologies due to infrastructure deficits, unhealthy policies, government instabilities, limited access to technology and education, and economic constraints [20-24]. Efforts must be made this early to ensure that African health care is not left behind in this AI revolution. In light of traditional development barriers, this may appear an aspirational goal. However, our data show a positive mindset toward embracing AI technology in health care, suggesting that it can be readily implemented. This is because Africa has a higher proportion of participants who believe in the usefulness of AI in health management and patient care currently (154/159, 96.9%) and in the future (147/159, 92.5%) and who are willing to support AI adoption (145/159, 91.2%) when compared to Europe (46/49, 93.9%; 38/49, 77.6%; and 41/49, 83.7%; respectively) and North America (114/130, 87.7%; 87/130, 66.9%; and 76/130, 58.5%; respectively). While further studies are needed to identify why AI adoption support is so low in North America, efforts should be made across all continents to enhance and accelerate AI adoption and to boost uptake and the quality and quantity of staff training programs.

Fewer female participants who completed the survey consider that AI has a role to play in health care management and patient practices (171/186, 91.9%) as against male participants (173/181, 95.6%). Moreover, a significantly lower proportion of female participants are willing to support AI adoption (123/186, 66.1%) compared to male participants (163/181, 90.1%; *P*<.005). We plan to conduct a qualitative study to determine the root cause of this difference. In the meantime, priority should be given to improve female participants’ awareness of the existence of AI and its usefulness, primarily by raising exposure rates of female participants to AI in schools, universities, hospitals, clinics, and community health facilities. We should also involve female participants in simulation exercises involving AI to improve their AI savvy and use. These practices may improve their acceptance and readiness to support AI adoption in health care management and patient care.

Key barriers to AI adoption as identified by our survey respondents are poor knowledge of AI (121/402, 30.1%), people’s fear of job loss (64/402, 15.9%), and staff resistance to change. Other leading barriers were the cost of acquisition and inadequate staff skills. Similar factor that hinders AI adoption were identified in previous studies [25-27]. When poor knowledge of AI and inadequate staff skills are combined, we have a disproportionately high ignorance-mediated barrier. All top barriers apart from the cost of acquisition are human resource–mediated, showing that appropriate communication, tailored training, and on-the-job exposure to AI can significantly reduce them. These barriers can further be reduced or removed through adequate explanation to patients and their families to gain social acceptance and build trust of AI technologies. Staff education and improved health literacy will most likely increase the willingness of health care workers to support AI adoption, acquisition, and installation.

This study achieved a notably high completion and participation rate compared with similar surveys, which often report substantially lower levels of engagement. This outcome was not unexpected, as we strategically leveraged the extensive professional and community networks of our research partners located in Africa (n=3), North America (n=3), South America (n=1), and Europe (n=2). In addition, the authors maximized their personal and professional connections to enhance outreach, while multiple reminders to the target population further improved response rates.

A particularly striking finding was that Black or African American participants demonstrated both a higher response rate and a more positive attitude toward AI adoption and use. Whether this reflects genuine readiness or aspirational enthusiasm remains an open question that warrants further investigation. Although several African nations, notably Rwanda, Nigeria, and Ghana [28-33], have demonstrated notable leadership in AI adoption relative to their regional peers, additional qualitative inquiry is needed to better contextualize and interpret this finding.

Taken together, the results underscore the importance of conducting more detailed qualitative studies to explore the underlying drivers of the variations observed in this research. A deeper, country-level analysis of AI adoption processes, rates, and patterns of use will provide a richer and more nuanced understanding of the current global landscape of AI adoption.

### Limitations

This study is subject to the typical limitations of a cross-sectional online survey, including selection bias, as participation was limited to individuals within the authors’ personal and professional networks. Additionally, the authors are unaware of the total number of individuals who received the questionnaire, making it impossible to calculate an accurate response rate. Furthermore, some email systems may block messages from unknown senders or redirect them to spam or junk folders, which could have further reduced participation.

To mitigate these limitations in future research, a more purposeful study design should be used—one in which participants are deliberately selected and actively encouraged to respond, thereby improving the likelihood of obtaining unbiased and representative results.

### Future Directions

Health care workers are aware of the AI revolution and recognize its usefulness in health care management and patient care. However, their knowledge, support, and adoption are each still low. Hence, there is a need to train more health care workers on not just the basic concepts of AI, but on AI use, algorithms, coding, and other technical issues. Universities, colleges, and institutions of higher education have a critical role in AI training and should be equipped and empowered to do this. In addition, a significant percentage of respondents were ignorant of those who were leading the AI adoption process—a pointer to communication failure or breakdown. Therefore, executive health care management should communicate better with their team to prevent innovation adoption failures. To effectively navigate the path forward toward realizing the potential of GenAI in health care, we recommend training health care leaders and workers in various aspects of AI management and use, provision of AI resources and infrastructure, and standardized oversight and guidelines [34]. This will play a significant role in building the health infrastructures required to achieve the United Nations Sustainable Development Goal 3 of “good health and well-being” [35].

In addition, we suggest that organizations and health care managers in Africa should deliberately and proactively invest in AI adoption and staff training, in order not to be left behind. Having health care workers who are favorable to AI adoption is a strength that African leaders can exploit right now. Individuals should be made to understand that AI works best when guided by skilled personnel and thus will not be replacing people. Although some jobs may be lost, AI will create new jobs and further opportunities that will balance the scale. To advance AI adoption, embedding, and use, governments should emulate Vietnamese leadership that has “identified AI as a key technology to boost its economy” and is taking deliberate and proactive steps to build partnerships, train engineers, pilot new products, support startups, and create an enabling environment for AI development to thrive [36,37]. As opined by one of the subject matter experts, “... successful AI implementation requires a combination of technical expertise, financial sustainability, and socio-political commitment. Each of these factors is crucial to fostering an environment conducive to AI adoption. All of these criteria are now met (in the private sector), but this cannot be said of the public system that is antiquated and overstretched by comparison” [36]. Yet, unlike the Vietnamese government, training in AI should go beyond engineers who develop the tools to include health care providers who adopt and use the tools to manage and care for patients. Funding public health institutions to adopt and embed AI will go beyond paying for the acquisition of AI and training of appropriate health care workers to expanding institutional infrastructure and mitigating staff resistance to change powered by fear of job loss and inadequate knowledge of AI functionalities and benefits.

In addition, various ethical concerns around data ownership, privacy, and use and the overall governance of AI need to be addressed by developing, adopting, and implementing organization-wide AI policies. As the said author also said, “data privacy, and the overall governance of AI technologies in healthcare, ... are critical for building public trust and ensuring the safe deployment of AI systems.” To achieve a seamless adoption of AI across the global health system, health care workers must be permitted unhindered oversight to alleviate inherent fear that may otherwise result in overt and covert resistance, which will thereby foster acceptance among patients [36].

Finally, to effectively navigate the path forward toward realizing the potential of GenAI in health care, we recommend training health care leaders and workers in various aspects of AI management and use, provision of AI resources and infrastructure, and standardized oversight and guidelines [34]. This will play a significant role in building the health infrastructures required to achieve the United Nations Sustainable Development Goal 3 of “good health and well-being” [35].

### Conclusions

This study reveals that AI adoption is a global phenomenon, with uptake increasing in all continents, including low- and middle-income countries. Our study documents the present adoption rates and some perceived factors that are facilitating or hindering AI adoption. In addition, health care workers believe that AI is useful and will be more relevant in the future. However, despite several advances in AI technology, deployment and adoption are still lower in some regions of the world. A significant number of participants are ready to support AI adoption and embedding in their organization, as they believe that AI will be very to extremely useful in health care management and practice.

## Appendix

Multimedia Appendix 1 Questionnaire.

Multimedia Appendix 2 CHERRIES checklist.

## Abbreviations

AI: artificial intelligence
CHERRIES: Checklist for Reporting Results of Internet E-Surveys
IRB: institutional review board

## Data Availability

Data are available on request from the author (OOO).
