# Supplementary material for: Global Adoption, Promotion, Impact, and Deployment of AI in Patient Care, Health Care Delivery, Management, and Health Care Systems Leadership: Cross-Sectional Survey
Source: J Med Internet Res. 2025 Oct 22;27:e70805. doi: 10.2196/70805 (PMC12590044; doi:10.2196/70805)
Supplement: Multimedia Appendix 1 [file jmir_v27i1e70805_app1.docx]

Artificial Intelligence adoption, embedding and deployment in Health & Management

Artificial intelligence (AI) is influencing every aspect of our lives, including how we live, work, teach, learn, lead, and provide patient care. While AI has been utilized in healthcare for over five years, there has been a lack of comprehensive study on its adoption, integration, and deployment within the sector. This cross-sectional study conducted across multiple continents aims to provide initial insights into these processes and inform future practices in healthcare.

Start of Block: Default Question Block

Q1 Do you think Artificial Intelligence (AI) has any role or usefulness in healthcare practice and management

- Yes (1)
- No (2)
- Neither true nor false (3)

Display This Question:

If Do you think Artificial Intelligence (AI) has any role or usefulness in healthcare practice and m... = Yes

Q2 If yes, how useful do you think AI is now in healthcare management and practice?

- Not at all useful (1)
- Slightly useful (2)
- Moderately useful (3)
- Very useful (4)
- Extremely useful (5)

Q2a In the future, how useful do you think AI will be in healthcare management and practice?

- Not at all useful (1)
- Slightly useful (2)
- Moderately useful (3)
- Very useful (4)
- Extremely useful (5)

Q3 Have you had any formal exposure or training in AI?

- Yes (1)
- No (2)
- Not Sure (3)

Display This Question:

If Have you had any formal exposure or training in AI? = Yes

| 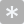 |
| --- |

Q4 If yes, what kind of training or exposure did you have (Tick all that apply)?

- Basic Orientation to AI (1)
- Training on AI use in patient care (diagnosis, treatment, lab services, etc.) (2)
- Training in AI use in management and leadership (3)
- Training in technical aspects of AI (4)
- Other forms of AI training (5)

Q5 Has your institution or organization adopted/begun the process of AI adoption, adaptation, and use?

- Yes, we have adopted AI (1)
- Yes, we will adopt AI (2)
- Yes, we are beginning to think about adopting AI (3)
- No, we have not started adopting AI (4)
- I do not know (5)

Display This Question:

If Has your institution or organization adopted/begun the process of AI adoption, adaptation, and use? != No, we have not started adopting AI

Q6 If your organization has started adopting AI, who is leading the process of AI adoption?

- Top-level/Executive Leadership (1)
- Middle Level/Management Staff (2)
- Operational Staff (3)
- Technical Staff (4)
- Outsourced (5)
- I do not know (6)

Q7 Has your organization trained anyone on AI use?

- Yes (1)
- No (2)
- I do not know/I am not sure (3)

Display This Question:

If Has your organization trained anyone on AI use? = Yes

Q8 If staff has been trained, who benefited from the training (Tick all that apply)?

- Executive Leadership/Top-Level (1)
- Management Staff/Mid-level (2)
- Operational Staff (3)
- IT staff (4)
- Others (5)
- I do not want to answer (6)

| 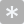 |
| --- |

Q9 Where do you think AI would be most useful in the healthcare industry in the coming years? (Select at most three (3) responses)

- Patient care (e.g. Treatment, Continuity of Care, Referral, etc.) (1)
- Diagnosis (e.g. Radiology, Pathology, Endoscopy, etc.) (2)
- Precision medicine (e.g. Gene Therapy, Cancer management, etc. (9)
- Leadership and management (3)
- Strategy development (8)
- Financial management (4)
- Resource management (6)
- Staff management (5)
- Report writing (7)
- Others (10)
- I do not want to specify (11)

| 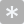 |
| --- |

Q10 Where else do you think AI would be very useful in the healthcare industry in the coming years? (Select at most three (3) responses)

- Patient care (e.g. Treatment, Continuity of Care, Referral, etc.) (1)
- Diagnosis (e.g. Radiology, Pathology, Endoscopy, etc.) (2)
- Leadership and management (3)
- Financial management (4)
- Staff management (5)
- Resource management (6)
- Report writing (7)
- Strategy development (8)
- Precision medicine (e.g. cancer management) (9)
- Others (10)
- I do not want to specify (11)

| 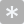 |
| --- |

Q11 Where do you think AI would be least useful in the healthcare industry in the coming years? (Select at most three (3) responses)

- Patient care (e.g. Treatment, Continuity of Care, Referral, etc.) (1)
- Diagnosis (e.g. Radiology, Pathology, Endoscopy, etc.) (2)
- Leadership and management (3)
- Financial management (4)
- Staff management (5)
- Resource management (6)
- Report writing (7)
- Strategy development (8)
- Precision medicine (e.g. cancer management) (9)
- Others (10)
- I do not want to specify (11)

| 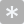 |
| --- |

Q12 What is/are the most important barrier(s) to AI adoption and implementation in your institution/industry (Select at most three (3) responses)

- Interest and attitude of staff (1)
- Staff resistance to change (2)
- Knowledge of AI (3)
- Staff skills and capacities (4)
- Cost of acquisition (5)
- Technology and equipment (6)
- Leadership and management (7)
- Organization wide adoption of AI (8)
- Fear of job loss (9)
- Others (10)

| 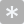 |
| --- |

Q13 What is/are the second most important barrier(s) to AI adoption and implementation in your institution/industry (Select at most three (3) responses)

- Interest and attitude of staff (1)
- Staff resistance to change (2)
- Knowledge of AI (3)
- Staff skills and capacities (4)
- Cost of acquisition (5)
- Technology and equipment (6)
- Leadership and management (7)
- Organization wide adoption of AI (8)
- Fear of job loss (9)
- Others (10)

| 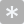 |
| --- |

Q14 What is/are the least important barrier(s) to AI adoption and implementation in your institution/industry (Select at most three (3) responses)

- Interest and attitude of staff (1)
- Staff resistance to change (2)
- Knowledge of AI (3)
- Staff skills and capacities (4)
- Cost of acquisition (5)
- Technology and equipment (6)
- Leadership and management (7)
- Organization wide adoption of AI (8)
- Fear of job loss (9)
- Others (10)

Q15 Will you support AI adoption and embedding in your organization?

- Yes (1)
- No (2)
- Not sure (4)

Display This Question:

If Will you support AI adoption and embedding in your organization? != Yes

Q16 If your answer to Q15 is no, maybe or not sure, why?

________________________________________________________________

Q17 In what other way(s) will AI be useful in the healthcare industry?

________________________________________________________________

End of Block: Default Question Block

Start of Block: Demographic Information: Now let us get to know you better.

Q18 What is your gender at birth

- Male (1)
- Female (2)
- Non-binary / third gender (3)
- Others (4)
- Prefer not to say (5)

Q19 How old are you in completed years?

- Less than 20 years (1)
- 20 - 29 years (2)
- 30 - 39 years (3)
- 40 - 49 years (4)
- 50 - 59 years (5)
- 60 years and above (6)

Q20 What is your highest educational qualification?

- High School Diploma/GED (1)
- Bachelors (2)
- Masters (3)
- Doctorate (4)
- Others (5)
- I do not want to specify (6)

Q21 How long have you been working in the health industry?

- Less than 5 years (1)
- 5 - 9 years (2)
- 10 - 14 years (3)
- 15 - 19 years (4)
- 20 - 24 years (5)
- 25 or more years (6)

Q22 In what organization are you currently working?

- Federal or State Government Facility/Institution (1)
- County/Local Government Facility/Institution (2)
- College/University (3)
- Non Profit/Public Charity (4)
- Private Facility/Institution (5)
- Others (6)

Q22a In which area of health have you worked or are currently working (Tick all that apply)?

- Health care services (1)
- Health education (2)
- Health informatics (3)
- Health technology (4)
- Health Administration/Management (5)
- Others (6)

Display This Question:

If In which area of health have you worked or are currently working (Tick all that apply)? = Others

Q22b If others, please specify

________________________________________________________________

Q23 What is your race or ethnicity

- Black/African American (1)
- White/Caucasian (2)
- Hispanic/Latino/Latinx (3)
- Native American/Alaska Native (4)
- Mixed (5)
- Pacific Island/Hawaii (6)
- Asian (7)
- Others (8)
- I prefer not to say (9)

Q24 In which continent are you currently located

- Africa (1)
- Europe (2)
- Asia (3)
- North America (4)
- South America (5)
- Australia (6)
- Others (7)

Q25 What did we miss? Please let us know

________________________________________________________________

End of Block: Demographic Information: Now let us get to know you better.

Start of Block: Contact Details

Q26 If you would like us to share the final report with you, please add your contact details/email:

________________________________________________________________

End of Block: Contact Details
